# Characterizing the Health Status of European Hake (*Merluccius merluccius*) in Areas with Different Anthropic Impacts (NW Mediterranean Sea)

**DOI:** 10.3390/ani16010014

**Published:** 2025-12-19

**Authors:** Irene Brandts, Sergi Omedes, Carmen Gilardoni, Marc Balcells, Montserrat Solé, Eve Galimany

**Affiliations:** 1 Institut de Ciències del Mar (ICM-CSIC), Pg. Marítim de la Barceloneta 37–49, 08003 Barcelona, Catalonia, Spain; irene.brandts@uab.cat (I.B.); omedes@icm.csic.es (S.O.); gilardonicarmen@gmail.com (C.G.); mbalcells@icm.csic.es (M.B.); msole@icm.csic.es (M.S.); 2 Department of Cell Biology, Physiology and Immunology, Autonomous University of Barcelona, Edifici M, Bellaterra, 08193 Cerdanyola del Vallès, Catalonia, Spain; 3 Institut Català de Recerca per a la Governança del Mar (ICATMAR), Pg. Marítim de la Barceloneta 37–49, 08003 Barcelona, Catalonia, Spain

**Keywords:** biomarkers, carboxylesterases, cholinesterases, parasites, marine litter, pollution

## Abstract

The high incidence of anthropogenic impacts in the Mediterranean basin raises concerns on the health and quality of commercial fish species. This study aims to evaluate the health status of the European hake, *Merluccius merluccius*, from three areas of the Catalan coast (NW Mediterranean Sea) with different anthropogenic impacts (i.e., chemical pollution, litter, …) and assess if hake could serve as a sentinel species. We measured biomarkers of chemical exposure including B-esterases, antioxidant enzymes (GST, GR, GPx, CAT), biotransformation markers (EROD), lipid peroxidation, and macro-parasite assemblages. Hake showed, generally, a good health status across all areas with homogeneous patterns for most parameters. Tissue-specific differences included elevated gonadal cholinesterases and higher brain and hepatic carboxylesterase activities in the south, and increased hepatic EROD but lower lipid peroxidation in the central Barcelona area. Parasite assemblages were dominated by Digenea, Cestoda, and Nematoda, with higher cestode prevalence in both central and south zones. In summary, despite a greater prevalence of environmental pollution in the central region, there was a homogeneous pattern in hake health indicators throughout the three studied fishing zones. These results establish a baseline for hake health in Mediterranean waters and suggest that the species’ high mobility and wide depth range may limit its utility to detect local-scale pollution impacts, though it may serve as a regional-scale bioindicator.

## Simple Summary

The Mediterranean Sea is subjected to several human-related (i.e., anthropic) impacts which alter the health of the organisms that inhabit it, including commercial fish like the European hake (*Merluccius merluccius*). This study assessed hake health along the Catalan coast (about 600 km in length) with varying pollution levels to determine if the species could serve as a local pollution sentinel. We evaluated hake general health status from three areas with different anthropic impacts by measuring different key factors: pollution exposure biomarkers (detoxifying enzymes), cellular stress/damage markers (antioxidant enzymes and lipid peroxidation), and macro-parasite assemblages. Results showed that hake were generally in good health across all areas, with most parameters being homogeneously distributed. Minor differences were observed in the central and south areas, where hake exhibited elevated levels of specific detoxifying enzymes and a higher prevalence of parasitic tapeworms compared to the north zone. The study establishes a baseline for hake health in the Catalan coast. However, the findings suggest that due to the species’ high mobility and wide depth range, the European hake may be limited in detecting local-scale pollution impacts. It is instead proposed as a useful regional bioindicator for monitoring the broader environmental quality of the Mediterranean Sea.

## 1. Introduction

The Mediterranean Sea, surrounded by countries with a combined population of about 571 million inhabitants in 2020, faces multiple anthropogenic stressors and it is considered one of the marine areas most impacted by human activities [1,2]. Population concentration near coastlines—with 25% of the coastal cities located within 5 km of the seashore—intensifies anthropic pressures, including fishing, climate change, chemical pollution, and marine litter. All these environmental pressures affect organisms inhabiting the Sea, including commercial fish species. Consequently, metals, polycyclic aromatic hydrocarbons (PAHs) and microplastic have been detected in target fish from the Mediterranean Sea, such as *Sardina pilchardus*, *Mullus* spp., *Engraulis encrasicolus*, or *Merluccius merluccius* [3,4,5].

The European hake, *M. merluccius* (Linnaeus, 1758), is a nektobenthic fish species widely distributed in the north-eastern Atlantic Ocean and the Mediterranean Sea [6]. In the western Mediterranean, hake is one of the most important target species for commercial fisheries, with hake landings in the Catalan coast (NW Mediterranean Sea) adding up to 577 tons in 2023, with most of them (90%) being caught by the Catalan bottom trawling fleet [7]. Given its commercial importance, broad distribution, and documented exposure to pollutants, including microplastics [8] and plastic additives accumulated in fish in muscle [1], hake has been proposed as a potential sentinel species in the Mediterranean Sea among other regions, such as the Basque continental shelf [9]. Pollution monitoring surveys conducted by EU state members aim to achieve and maintain a good environmental status (GES) within the Marine Strategy Framework Directive [10]. Sentinel organisms are a type of health indicator used to signal environmental health alerts on a particular ecosystem [11]. Hake has a demersal–pelagic behavior and a wide depth range (30–1000 m) distribution, providing integrated exposure across environmental compartments [12], with its high trophic position enabling contaminant biomagnification. Its large body size allows multi-tissue biomarker assessment, and its commercial value ensures cost-effective sampling through existing fisheries monitoring programs [6,9,13]. However, the suitability of hake as a sentinel species to detect spatial differences in pollution at local scales requires empirical validation. Several parameters can be used to study the health of aquatic organisms, including those used as sentinel species. Biomarkers are measurable indicators at the sub-organism level that provide valuable information to assess the health status of organisms [14]. The measurement of biomarkers as an indirect approach to monitor chemical pollution is especially adequate when addressing mixtures that require complex methodologies [15,16]. Among the biomarkers selected, those encompassing enzymes involved in the detoxification of xenobiotics and their metabolites stand out. A relevant family of such enzymes are B-esterases, previously validated as biomarkers of pesticide exposure in terrestrial and aquatic species, ranging from mammals to reptiles, birds, and fish [16,17,18,19]. On the other hand, the characterization of the parasitic load in a chosen species, e.g., monitoring transmitted helminth endo-parasites, has also proven to be valuable pollution bioindicators [20]. The presence of parasites can also modify biomarker responses since they are natural stressors that have detrimental effects in their hosts [21]. In this study, integrating biomarkers across biological levels, i.e., B-esterases and EROD for exposure [16,17,18,19], antioxidant enzymes and lipid peroxidation for oxidative stress [22], and parasite assemblage and condition index as a reflection of general health [20], seeks to provide a comprehensive assessment of the fish health in contaminated environments, unattainable through single-parameter approaches.

The Catalan coast (NW Mediterranean Sea) presents a suitable study area due to well-documented gradients in anthropic impacts, with the Barcelona metropolitan area experiencing the highest pollution loads from industrial, urban, and maritime sources, while northern and southern zones face lower but distinct pressures [23,24,25,26,27,28]. Then, the aim of this study was to evaluate whether the European hake can serve as an effective sentinel species by assessing its health status across areas with contrasting anthropic impacts along the Catalan coast. Specific objectives were (1) to undertake a holistic assessment of the fish health status through condition indices, biochemical biomarkers and parasite incidence; (2) to establish correlations, if any, between health status indicators in the brain, kidney, gonads, liver and muscle; and (3) to compare the hake health status of the studied specimens in the three fishing grounds of the Catalan coast with different levels of anthropic impacts.

## 2. Materials and Methods

### 2.1. Sampling Zones

**Figure 1:**
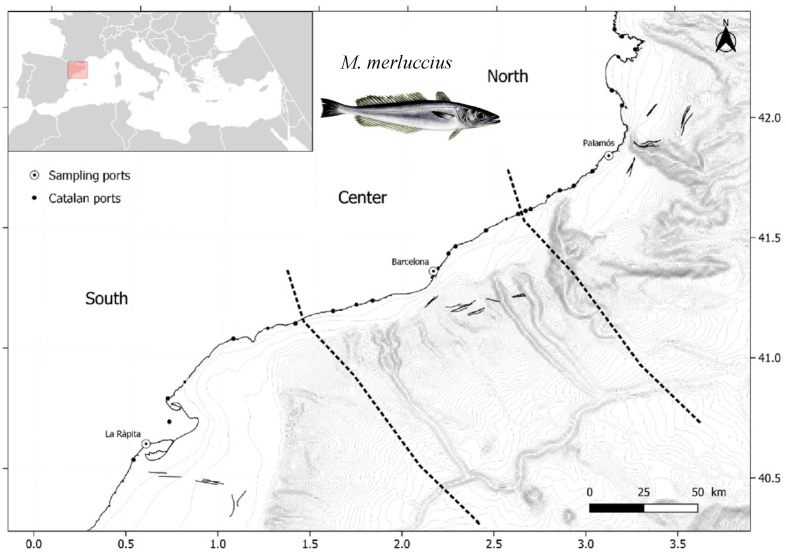
Sampling map of the three fishing grounds separated by dashed lines (i.e., north, center, south) in the NW Mediterranean region where hake (*Merluccius merluccius*, picture included in the map) were sampled. Black short lines are the sampled hauls in each zone.

The study was carried out along the Catalan Coast, in the NW Mediterranean Sea (Figure 1). The coast is 580 km long and sampling was designed by dividing the area into three zones, according to their province borders and different anthropic influence. From each zone, one port of main commercial importance was selected: (i) north zone (41°48.360′, 3°15.230′; average depth 126 m), including the port of Palamós, (ii) center zone (41°13.961′, 2°8.628′; average depth 109 m), including the port of Barcelona, and (iii) south zone (40°28.269′, 0°57.292′; average depth 73 m), including the port of La Ràpita. The center zone includes the great metropolitan area of Barcelona, with 5.8 million inhabitants in 2023, therefore exhibiting the highest contamination due to anthropogenic impacts. In the Levantine–Balearic demarcation, monitoring of organochlorine compounds in marine sediments revealed spatial variability linked to anthropogenic pressures, detecting the highest concentrations of PCBs (polychlorinated biphenyls) and DDT, a persistent organic pollutant, near the metropolitan area of Barcelona [24]. Furthermore, the Barcelona area has also shown the highest concentrations of both trace metals and organic pollutants in monitoring studies along the Catalan inner continental shelf [25,26]. Moreover, Balcells et al. (2023) [27] found the greatest densities of benthic marine litter in the Barcelona area (13.75 ± 3.25 Kg Km^−2^), with plastic (74.3%) being the most common type of waste. The area holds one of the busiest airports and ports, characterized by high levels of tourism and significant industrial development. In contrast to the center zone, the north and south zones are characterized by low population densities, therefore being less affected by industrialization and urbanization, especially in the northern zone. As a result, lower densities of marine litter were observed, with recorded values of 5.35 ± 0.89 Kg Km^−2^ in the northern zone and 5.86 ± 1.04 Kg Km^−2^ in the southern zone [27]. The south zone includes the Ebre Delta, one of the largest wetlands in the Mediterranean region, designated as a Natural Park in 1983. Despite its relative isolation from major urban centers, the south zone is surrounded by rice crops and other agricultural activities. In the past, these agricultural practices involved the use of insecticides such as DDT for pest control. Although their use has since been banned or heavily restricted, their chemical stability allows them to persist in the environment [28]. This is consistent with a recent report published by the Spanish Government, where high concentrations of DDT in sediments at the mouth of the Ebre River were reported, reflecting its persistence in marine sediments [24]. These zones represent three complementary scenarios—urban–industrial, low-impact, and agricultural contamination—enabling the evaluation of hake health responses to documented pollution gradients.

### 2.2. Fish Sampling and Morphometric Measurements

Individual hake specimens were fished in September 2022 by commercial bottom trawlers registered at the three ports previously described; 10 individual fish from each zone were sampled. Male hake individuals were selected to avoid potential hormonal influences associated with the female reproductive cycle in the biochemical analyses (size range 25–35 cm in length). On board, fish were immediately frozen in dry ice and transported to the laboratory where brain, kidney, liver, gonads and a portion of muscle (1 × 1 cm, always from the dorsal region under the first dorsal fin) from fish were excised and stored at −80 °C for further analysis. The samplings were all performed simultaneously in time, to avoid differential influence of physical water parameters modulating biomarker responses in hake [29]. For parasitological studies, 20 additional fish from each zone were captured in May 2023 and transported in coolers to the laboratory. All fish were measured (total length (TL), cm) and weighed (±0.1 g). These biometric data were used to calculate Fulton’s condition factor (K) as follows:

K = weight/(length) ^3^ × 100

The liver and gonads were also weighed (±0.01 g); the values were used to calculate the hepatosomatic index (HSI) or gonadosomatic index (GSI) as follows [30]:

HSI or GSI = (liver or gonad weight)/(total weight) × 100

### 2.3. Tissue Preparation for Biochemical Analyses

Brain, kidney, gonads and a portion of muscle were homogenized using ice-cold buffer K-phosphate (50 mM pH 7.4) containing 1 mM ethylenediaminetetraacetic acid (EDTA) in a 1:5 (*w*:*v*) ratio using a Polytron^®^ (Polytron, Duluth, GA, USA). Liver was homogenized in a similar manner but in a 100 mM phosphate buffer which also contained 150 mM KCl, 1 mM dithiothreitol (DTT), 0.1 mM phenanthroline, 0.1 mg/mL trypsin inhibitor and the ratio was 1:4 (*w*:*v*). After homogenization, a 200 µL aliquot was separated from the liver, for lipid peroxidation analysis. Then, all homogenates were centrifuged at 10,000× *g* 30 min at 4 °C to obtain the supernatants, or post-mitochondrial S9 fraction. Approximately 1 mL of each S9 sample was aliquoted and stored at −80 °C for further enzymatic determinations. Liver post-mitochondrial fraction (S9) was further processed to obtain further subcellular fractions, by centrifuging at 100,000× *g* 60 min at 4 °C. The resulting supernatant was separated as the cytosolic fraction and the remaining microsomal pellet was dissolved in homogenization buffer, with an additional 20% glycerol, in a 2:1 (*w*:*v*) ratio [31]. Microsomal and cytosolic fractions were also stored at −80 °C for further biochemical determinations.

### 2.4. Biochemical Determinations

All assays were performed in triplicate at 25 °C except EROD (30 °C) using an Infinity Pro200 TECAN (Männedorf, Switzerland) spectrophotometer as plate reader using the Magellan v6.0 kinetic mode.

#### 2.4.1. Protein Determination

Protein content, to which enzyme activities were referred, was determined using the Bradford method [32] adapted to microplates, using the Bio-Rad Protein Assay reagent (Hercules, CA, USA). Total protein quantification for each of the samples was performed at 495 nm using an external standard curve with bovine serum albumin (0.05–0.5 mg/mL).

#### 2.4.2. Determination of B-Esterases Activities

The cholinesterase (AChE, BChE, and PrChE) and carboxylesterase (pNPA-CE, pNPB-CE, αNA-CE and αNB-CE) activities were measured in the S9 fraction in liver, muscle, kidney, gonads, and brain of hake. To determine ChE activities, Ellman’s protocol was followed [33], using acetylthiocholine iodide (ASCh), butyrylthiocholine iodide (BSCh) and propionylthiocholine iodide (PrSCh) as substrates for each of the corresponding enzymes (AChE, BChE and PrChE). A 96-well transparent plate was used (Nunclon, Thermofisher, Waltham, MA, USA); mix 150 µL of phosphate buffer (50 mM, pH = 7.4) containing DTNB (5,5′-dithio-bis-(2-nitrobenzoate)) at 0.27 mM with 25 µL of sample in each well, incubate for 2 min and then initiate the reaction by adding 50 µL of the respective substrates, each at 1 mM final concentration. The reaction was monitored for 5 min at 412 nm. Activities of CEs were measured using as proxy the hydrolysis rates of four commercial substrates: p-nitrophenyl acetate (pNPA) and p-nitrophenyl butyrate (pNPB), α-naphthyl acetate (αNA), and α-naphthyl butyrate (αNB). In each microplate well, 25 µL of sample was mixed with 200 μL of phosphate buffer containing the corresponding substrate at a final concentration of 1 mM, for pNPA and pNPB [34], or 0.250 mM, for αNA and αNB [35]. As described by these methodologies, the formation of p-nitrophenol was monitored at 405 nm and the naphthol formation at 235 nm, for 5 min. Enzymatic activities were expressed as nmol/min/mg protein.

#### 2.4.3. Antioxidant Enzymes and Lipid Peroxidation (LPO) in Liver

Catalase (CAT) activity was measured using H_2_O_2_ (50 mM) as substrate and the decrease in absorbance was read at 240 nm and expressed in μmol/min/mg protein [36]. Total glutathione peroxidase (GPX_tot) and selenium-dependent glutathione peroxidase (GPx_Se) were determined using cumene hydroperoxide (CHP, 0.625 mM) and H_2_O_2_ (2.81 mM) as respective substrates [37]. GPx activity was determined measuring NADPH hydrolysis. Glutathione reductase (GR) activity assay used oxidized glutathione (GSSG, 0.91 mM) as substrate and measured NADPH hydrolysis. Glutathione S-transferase (GST) determination was performed using 1 mM GSH as substrate [31]. GPx_tot, GPx_Se, GR and GST activities were all read at λ = 340 nm and are expressed in nmol/min/mg protein. All these antioxidant enzymes were measured in the cytosolic fraction of the liver homogenates. The LPO levels (LPO) were quantified using an adaptation of the 1-methyl-2-phenylindole colorimetric method [38]. Liver homogenates were incubated for 40 min at 45 °C with 1-methyl-2-phenylindole at a final concentration of 65 µM in the presence of 150 µL of HCl (37%). After incubation, the reaction was stopped by placing the samples in ice and samples were centrifuged at 10,000× *g* for 10 min at room temperature. Supernatant was read at 586 nm in a microplate reader. LPO quantification was performed using an external standard curve with 1,1,3,3-tetramethoxypropan (0–10 µM) and results are expressed in nmol MDA/g *w*/*w*.

#### 2.4.4. CYP1A1 EROD Activity

Phase I biotransformation CYP1A1-dependent 7-Ethoxyresorufine-O-deethylase (EROD) activity was based on the fluorometric kinetic method following the methodology described in Solé et al. (2012) [39]. In brief, 50 μL of microsomes was incubated in each well with the substrate, 7-ethoxyresorufin (2 μM), and the cofactor NADPH (at 200 μM, respectively), at 30 °C for 10 min. A calibration curve (range 0–160 nM) for each specific metabolite was performed and the increase in fluorescence was read, at 537/583 nm excitation/emission [34]. Activities are expressed in pmol/min/mg protein.

#### 2.4.5. Lactate Dehydrogenase (LDH) in Muscle

The measurement of the anaerobic metabolism pathway was assessed as LDH activity in muscle S9 following the methodology described by Vassault et al. (1983) [40], with some adaptations. A 96-well plate was used, with 0.2 mM NADH and 1 mM of pyruvate as the final concentration in each well. The reaction was read at 340 nm for 5 min and LDH activity expressed was as nmol/min/mg protein.

#### 2.4.6. Parasitological Study

When each fish was dissected, the following tissues were checked for the potential presence of parasites: body surface, fins, eyes, gills, branchial and body cavities, and viscera (i.e., stomach, intestine, heart, spleen, liver, gonads, kidney, swimming bladder and mesenteries). The tissues were observed under a stereoscopic microscope and the musculature was observed between two transparent plates with light emitted from below. Parasites, when found, were collected using thin dissecting forceps. Specimens were observed by an optic microscope and determined using specialized bibliography [41,42,43,44,45]. Parasitological indices such as prevalence, defined as the percentage of hosts infected divided by the number of examined hosts, and mean abundance of infection, defined as the number of parasites divided by the number of examined hosts, were calculated for each taxonomic group in each zone following Bush et al. (1997) [46]. In addition, for each fish host, the parasitic load was calculated as the total number of parasites of all taxonomic groups. The biological parasite diversity indexes (species richness (S), Shannon–Wiener diversity (H), and evenness (E) and similarity indexes (Sorensen quantitative and qualitative) were calculated following Magurran (2004) [47]. The qualitative Sorensen index evaluated the average proportion of shared species between two zones in detail whereas the quantitative Sorensen index evaluated the proportion of similarity regarding the abundance of the shared species.

### 2.5. Statistics

Statistical analyses for morphometric measurements and biochemical analysis were performed using the software R (ver. 4.1.3) (R Core Team (Vienna, Austria), 2022), with the following packages: car (ver. 3.0), dplyr (ver. 1.0.8), rstatix (ver. 0.7.0), broom (ver. 0.8.0), corrplot (ver. 0.92), Hmisc (ver. 4.6). Firstly, normality and equality of variance were checked using Shapiro–Wilk and Levene’s tests, respectively, and data were transformed when necessary. The weight, length and condition index were compared among zones using Analysis of Variance (ANOVA). Analysis of Covariance (ANCOVA) tests were performed on all B-esterases and oxidative stress parameters, to consider differences in weight and length between groups. Tukey’s multiple comparison test was applied as a post hoc, when appropriate. Pearson correlations and principal component analyses (PCAs) were performed in liver, to explore relationships between parameters and cellular fractions. For parasitological analysis, prevalence and abundance calculated following [46] for the three most predominant taxonomic groups (Cestoda, Nematoda and Digenea); the total abundance, species richness and Shannon–Wiener index were tested among zones using Generalized Lineal Models (GLMs). In all models, the effect of hake length was evaluated as a covariable and it was discarded because it was non-significant. Parasite prevalence was modeled with binomial distribution, while abundance, total abundance, species richness and Shannon–Wiener index were modeled with Poisson distribution. Models were corrected for overdispersal when it was necessary.

## 3. Results

### 3.1. Condition Factor and Gross Morphometric Measurements

Morphometric measurements are shown in Table 1. The weight of hake caught in each of the three areas was significantly different among all groups (F(2,27) = 23.10; *p* < 0.0001), with hake from the southernmost area being smaller (north vs. center, *p* = 0.045; north vs. south, *p* = 0.0007; center vs. south, *p* < 0.0001). Length also showed significant differences among groups (F(2,27) = 45.08; *p* < 0.0001), with hake from the southernmost area being shorter (north vs. south, *p* < 0.0001; center vs. south, *p* < 0.0001). However, the condition factor (K) and hepatosomatic index (HSI) did not vary between fish from the three fishing grounds (*p* > 0.05). The gonadosomatic index (GSI) was also lower in southern specimens when compared to the center areas ((F(2,27) = 10.30; *p* = 0.0005); north vs. south, *p* = 0.0015; center vs. south, *p* = 0.0014). Table S1 shows the details of the statistical analyses.

### 3.2. Biochemical Determinations

#### 3.2.1. B-Esterase Activities in Hake Tissues

**Figure 2:**
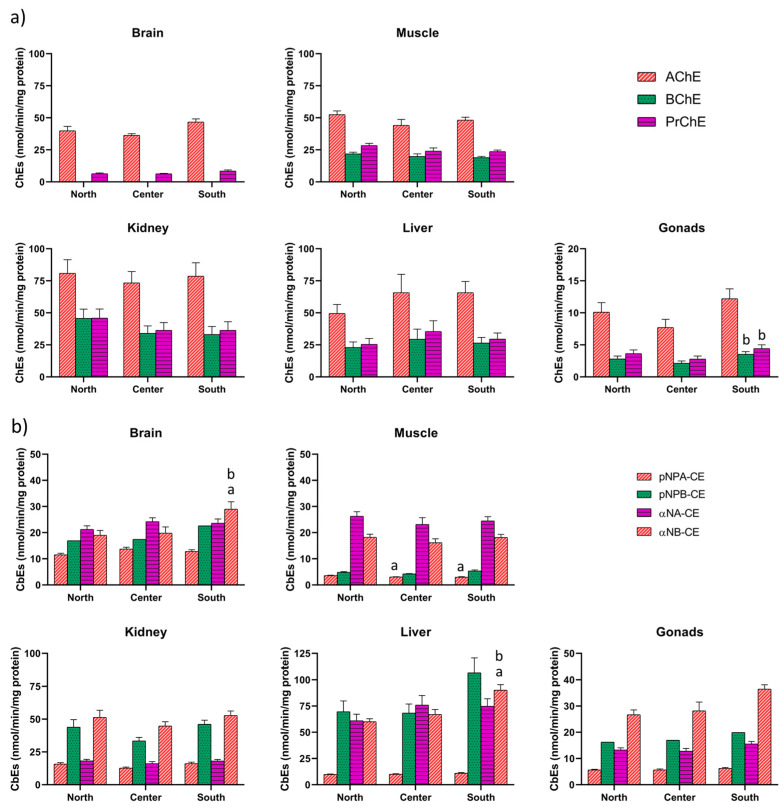
Activities of (**a**) cholinesterases and (**b**) carboxylesterases in several tissues of hake *(Merluccius merluccius)* from three fishing grounds in the studied area (NW Mediterranean Sea). Values are presented as mean ± standard error of the mean (SEM). Statistical differences are marked as follows: a vs. north, b vs. center (*p* < 0.05).

The activities of B-esterases per tissue and fishing zone corresponding to the seven assayed substrates are shown in Figure 2. Figure 2a reports the hydrolysis activities for ChEs (AChE, BChE, PChE) in fish from the three different fishing zones. No significant differences were found in ChE activities in the brain, muscle, kidney and liver. In gonads, both BChE and PChE activities showed differences between groups (F(2,26) = 5.7956, *p* = 0.008 and F(2,25) = 6.047, *p* = 0.007, respectively). Both enzymes were significantly higher in fish from the south zone with respect to central zone (BChE *p* = 0.006 and PChE *p* = 0.005), but no other significant differences were found. Results from CE determinations in the selected tissues are indicated in Figure 2b, corresponding to four different substrates as indicative of putative CE isoforms. No differences between the three fishing zones were found in any of the CEs activities in kidney nor in gonads. In muscle, pNPA-CE activity was higher in hake from the north with respect to both center (*p* = 0.002), and south (*p* = 0.009). In the brain, αNB-CE activity showed differences between groups (F(2,27) = 5.3606, *p* = 0.01), with values significantly lower in hake from north and center, with respect to fish from the south (*p* = 0.017 and *p* = 0.030, respectively). Likewise, hepatic αNB-CE activity also showed differences between groups (F(2,26) = 5.9273, *p* = 0.007), with lower activity in both northern and central fish, with respect to southern zone (*p* = 0.002 and *p* = 0.0001, respectively). No other significant differences were found between groups. Table S2 shows the details of the statistical analyses.

#### 3.2.2. Antioxidant Enzymes and Lipid Peroxidation (LPO) in Liver

Biochemical parameters related to oxidative stress measured in liver of hake are shown in Table 2. The levels of the enzymatic antioxidant defenses such as GST, GR, GPx_tot, GPx_Se and CAT did not vary between hake from the three fishing grounds.

#### 3.2.3. CYP1A1 EROD Activity

EROD activity was significantly higher in fish from the central zone when compared to the south fish (*p* = 0.020), and no other significant differences were found. On the other hand, hepatic LPO values were lower in fish from the central zone, when compared to north (*p* = 0.03) and south (*p* = 0.005) zones.

#### 3.2.4. LDH in Muscle

LDH activity (in nmol/min/mg prot) was similar at the three sampling zones, i.e., north (4311.9 ± 230.6), center (4194.1 ± 152.2) and south (3485.4 ± 327.6), with no statistical zone differences (*p* > 0.05).

### 3.3. Parasitological Study

A total of eight parasite species were recorded, which belonged to six different taxa, i.e., Monogenea, Digenea, Cestoda, Nematoda, Acanthocephala, Copepoda (Table 3; Figure S2). The endo-parasites Digenea, Cestoda and Nematoda were present in the three zones. Prevalence and abundance of Digenea and Nematoda had no significant differences among the three zones (*p* > 0.05). However, significant differences were observed for Cestoda in which prevalence and mean abundance were lower in the north region (*p* = 0.004, *p* = 0.005, respectively). The other identified parasite taxa had very low prevalence and could not be compared among zones (Table 3).

Total abundance, species richness and Shannon–Wiener diversity index did not show significant differences among zones (*p* > 0.05). The Sorensen similarity index for qualitative data, representing the number of shared species, was high (55–80%) between zones. The Sorensen similarity index for quantitative data (Figure S1) representing the abundance of shared species was low and similar between zones (10–18%).

### 3.4. Correlations and PCA

**Figure 3:**
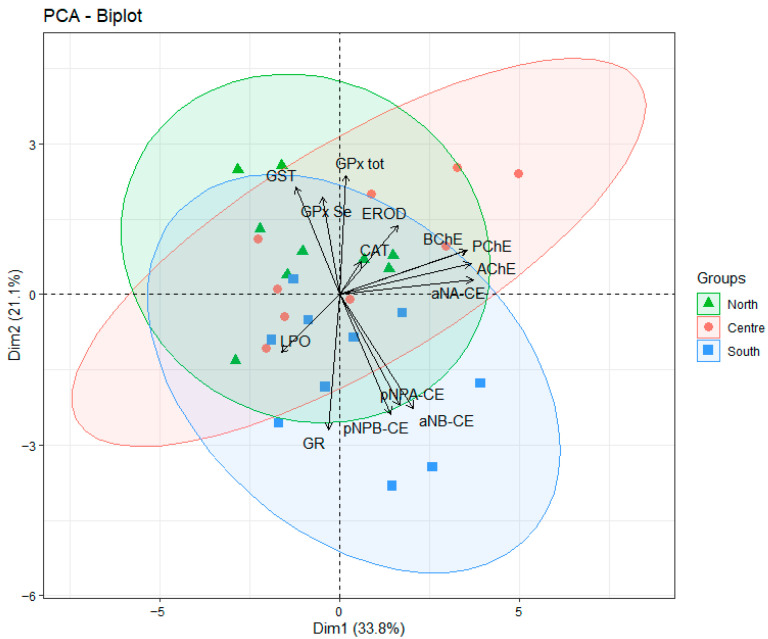
PCA analysis of 14 biomarkers measured in hake (*Merluccius merluccius*) liver from the three fishing grounds in the studied area (NW Mediterranean Sea). Variables included cholinesterases (AChE, BChE, PrChE), carboxylesterases (pNPA-CE, pNPB-CE, aNA-CE, aNB-CE), antioxidant enzymes (GST, GR, GPx_tot, GPx_Se, CAT), biotransformation enzyme (EROD), and lipid peroxidation (LPO).

A two-dimensional PCA plot that includes the B-esterase and oxidative stress measurements, all from the liver, is shown in Figure 3 using individual fish as replicate samples (n = 10 per zone) for each of the three sampling zones. A total of 54.9% of variance can be explained by these first two dimensions (Dim1 and Dim2). Replicates from different zones show substantial overlap, indicating a lack of distinct clustering based on the zone factor.

## 4. Discussion

In the present study, we formulated a multibiomarker approach to see if the pollution status of the different fishing areas could be reflected in general fish health. Our results suggest that, despite a greater prevalence of environmental pollution in the central region—near the Barcelona metropolitan area—and some parameters differing significantly, there was no consistent pattern correlated with the pollution gradient. Therefore, there was a mostly homogeneous pattern in hake health indicators throughout the three studied fishing zones based on parameters such as the condition index (K), biochemical markers and parasite incidence. Similar multibiomarker approaches have been used to monitor pollution generated from a broad variety of sources and chemical mixtures in multiple key commercial species, i.e., red mullet (*Mullus surmuletus*), bogue (*Boops boops*), and anchovy (*Engraulis encrasicolus*) [22,48,49,50,51].

Our results revealed that most health indicators showed homogeneity across the three fishing zones, with similar condition factor (K), hepatosomatic index (HSI), and most biomarker activities. Some specific parameters showed regional differences, i.e., elevated gonadal cholinesterases and carboxylesterases in brain and liver in the south, higher muscle pNPA-CE in the north, elevated hepatic EROD activity in the center (Barcelona) area, lower lipid peroxidation in this same area and lower cestode prevalence in the north. However, these differences did not follow a consistent pattern aligned with the documented pollution gradient, where the Barcelona metropolitan area exhibits the highest contamination loads [24,25,26,27].

The adopted multibiomarker approach included parameters indicative of neurotoxicity, detoxification processes, oxidative stress, energy metabolism and parasite incidence. Biomarker measurements such as the study of CEs isoforms have been used to monitor anthropogenic chemicals in different marine species, such as seabass (*Dicentrarchus labrax)* [52], sardine *(Sardina pilchardus*) [53] and different tuna species [54]. In addition, the measured antioxidant enzymes, i.e., GR, GPx_total, GPx_Se, catalase, and lipid peroxidation (LPO), have previously been shown to reflect plastic-related pollution in fish [22]. However, in the present study, the majority of assessed biomarkers showed no clear differential trend between fishing zones, and the few significant differences observed were tissue-specific and did not relate consistently with pollution levels. The PCA, which was used as a multidimensional tool to give an integrative perspective of the results, confirmed that there was a lack of zone differences among the studied specimens, when considering hepatic biomarkers.

Despite the overall homogeneity in health indicators, the elevated hepatic EROD activity in hake from the Barcelona area provides evidence of exposure to organic contaminants. EROD activity, catalyzed by cytochrome P450 1A1 (CYP1A1), is a highly specific biomarker of exposure to aryl hydrocarbon receptor (AhR) agonists, particularly polycyclic aromatic hydrocarbons (PAHs) and dioxin-like polychlorinated biphenyls (PCBs) [24,25,55,56]. The Barcelona metropolitan area is well-documented as having elevated concentrations of both PAHs—primarily from shipping activities, oil pollution, and urban runoff [55,56]—and PCBs in sediments [24]. The EROD induction observed in our study is consistent with this contamination gradient and demonstrates that hake indeed exposed these pollutants. Moreover, accumulation of pollutants such as PAHs, heavy metals, PCBs and plastic particles in hake specimens from the Mediterranean have previously been reported, with different pollution levels showing specificity to sites, finding greater concentrations of PAHs in hake fish in the surroundings of Barcelona [56,57,58]. Nevertheless, the increase in EROD as a biomarker of exposure was not accompanied by evidence of oxidative stress (no differences in GST, GR, GPx, CAT) or LPO damage. In fact, LPO levels were significantly lower in the Barcelona zone compared to north and south zones. This pattern suggests that hake possess robust detoxification capacity capable of metabolizing contaminants without progression to oxidative damage.

The fact that the parasite assemblage was also relatively homogeneous among the studied zones suggests that the physicochemical characteristics of the three fishing grounds do not affect the transmission of parasites. The three endo-parasite taxa that were dominant in all zones (Digenea, Cestoda and Nematoda) are reliable bioindicators of good environmental status, as their complex life cycles require the integrity of the trophic chain [20]. The European hake is the definitive host for the Digenea and Cestode but a parenthetic host for Nematoda [59]; however, if anthropogenic factors disrupted hosts involved in the life cycle, parasite population would respond accordingly. The exception is the higher cestode prevalence in central and southern areas when compared to the north. This pattern could reflect pollution-induced immunosuppression, as exposure to PAHs, PCBs, and legacy pesticides can compromise fish immune defenses, increasing susceptibility to parasitic infections [60]. This would be consistent with the elevated EROD activity in Barcelona, indicating significant contaminant exposure. Alternatively, anthropogenic activities may alter intermediate host communities with urban organic enrichment (center) or agricultural runoff (south) potentially favoring certain crustacean species and thereby enhancing parasite transmission. However, without direct measurements of fish immunocompetence or characterization of intermediate host communities, we cannot definitively distinguish between pollution-mediated effects and natural spatial variation in prey assemblages. The fact that overall parasite diversity and abundance remained similar across zones suggests that any pollution effects on parasite communities are subtle, and agrees with former observations in hake in which the total parasite prevalence and abundance was also similar in the three fishing grounds [61].

Monitoring the status of the ocean can be performed using sentinel species that should meet certain criteria, i.e., being easily identifiable, having a broad geographical distribution, being abundant and accessible, and having good knowledge of its biology and ecology [62]. Hake meets all these criteria and, additionally, it is a valuable commercial species. Giani et al. (2019) [57] proposed hake to be used as a small-scale bioindicator of plastic pollution, finding different ingestion rates in two fishing geographical sub-areas (GSAs), with microplastics ingested in 48% of the studied fish in the Ionian Sea (GSA-19) but only an occurrence of 8.3% in the North Tyrrhenian Sea (GSA-9). In the present study, while the fishing grounds from the center (Barcelona) area contain much larger quantities of marine litter and documented higher concentrations of chemical contaminants than the other studied zones [24,25,26,27], hake health indicators did not consistently reflect this pollution gradient. Similarly, the agriculture-related products released from the crops in the south zone do not appear to have a direct impact on hake health, with the parameters presented in this study. This disconnect between documented exposure and health effects suggests that hake may not be suitable for local-scale pollution monitoring.

The lack of consistent pollution–response patterns in hake could be explained by the species’ biological and ecological characteristics. Hake is a major component of the demersal fish assemblages, reaching depths from 30 to 1000 m [12]. The species’ wide depth range means that hake integrate contamination from various environmental compartments, such as surface waters, water columns, and sediments. It is also a highly mobile species capable of performing seasonal and vertical migrations [63], which facilitate the integration of environmental signs over broad spatial scales. Therefore, fish caught in a specific fishing ground may represent a mixture of exposure histories from multiple areas, diluting local pollution signatures.

Traditionally, the assessment of chemical exposures in the marine environment is undertaken using sessile and sedentary organisms inhabiting coastal environments, such as mussels *Mytilus galloprovincialis* and mullets *M. barbatus* [64], both considered pilot organisms in pollution biomonitoring programs across the Mediterranean basin (MEDPOL, 1986). The bivalve *M. galloprovincialis* is a sessile bivalve and readily accumulates contaminants such as heavy metals and hydrocarbons whereas *M. barbatus* is a benthic, sediment-associated feeding fish, widely used due to its coastal distribution, commercial importance, and well-characterized biology [64]. These species are effective local-scale sentinels precisely because of their limited mobility and site-specific exposure. However, the predominant reliance on sedentary organisms in marine monitoring programs overlooks the impact at broader spatial scales [65]. Predatory pelagic fish could serve as valuable sentinel species, given their crucial ecological role at higher trophic levels and their influence on ecosystem sustainability [54]. Thus, their use may reflect monitoring marine pollution at a global scale due to factors such as their wide-ranging movements and complex exposure patterns, as the present study reflects, in this relevant commercial species. Thus, hake and sedentary sentinels could be used as complementary rather than redundant monitoring functions: sessile species like mussels detect local pollution hotspots and acute contamination events, while mobile species like hake integrate regional contamination patterns and assess ecosystem-level effects across larger geographic areas. The present study suggests that hake may be more suitable for basin-scale or regional pollution assessment in the Mediterranean rather than for distinguishing between neighboring fishing grounds with different local impacts.

## 5. Conclusions

The European hake (*M. merluccius*) studied showed a homogeneous health status across areas with different anthropic impacts. While specific biomarker responses—particularly elevated EROD activity in the Barcelona area—may indicate exposure to anthropic chemicals, these responses did not translate into generalized health impairment or follow consistent patterns aligned with documented pollution gradients. The species’ high mobility, wide depth range, and detoxification capacity most likely limit its utility as a sentinel for local-scale pollution monitoring, as individuals integrate environmental signals across broad spatial scales. However, the present results can establish a baseline for hake health in the region and may also be used to set a baseline for the health condition of hake in Mediterranean waters. Future monitoring programs may use a combination of both, sedentary organisms (for local-scale, site-specific assessment) and mobile species like hake (for regional integration), to capture pollution impacts across multiple spatial scales. Moreover, the fact that our data show generally homogeneous good health in the NW Mediterranean Sea for hake is a positive outcome, considering humans as final consumers of this fish species.

## Figures and Tables

**Table 1:** Biometric indicators determined in hake (*Merluccius merluccius*) from three different fishing grounds of the Catalan coast. Values are presented as mean ± standard error of the mean. When values have different letters, it indicates statistical differences between the different locations (One-way ANOVA, *p* < 0.05). K = Condition factor, HSI = hepatosomatic index, GSI = gonadosomatic index. Calculations are detailed in the Methods section.

Location	Length (cm)	Weight (g)	Condition Factor (K)	Hepatosomatic Index (HSI)	Gonadosomatic Index (GSI)
North	28.6 ± 0.5 ^a^	158.8 ± 7.2 ^a^	0.67 ± 0.02 ^a^	2.53 ± 0.2 ^a^	29.19 ± 3.6 ^a^
Center	30.2 ± 0.8 ^a^	206.6 ± 21.2 ^b^	0.72 ± 0.02 ^a^	4.31 ± 0.7 ^a^	35.80 ± 9.1 ^a^
South	22.2 ± 0.5 ^b^	79.4 ± 6.0 ^c^	0.71 ± 0.01 ^a^	2.54 ± 0.2 ^a^	11.25 ± 3.6 ^b^

**Table 2:** Antioxidant enzymes and oxidative stress parameters in liver of hake (*Merluccius merluccius*) from three different fishing grounds of the Catalan coast. Values are presented as mean ± standard error of the mean. GST, GR, GPx_tot, GPx_Se and CAT in nmol/min/mg protein, EROD in pmol/min/mg protein and LPO in nmol MDA/g *w*/*w*. Different letters show significant differences in the different locations (ANCOVA, *p* < 0.05).

Biomarker	North	Center	South
GST	350.6 ± 26.2 ^a^	347.1 ± 32.2 ^a^	236.8 ± 12.2 ^a^
GR	6.9 ± 0.2 ^a^	6.7 ± 0.6 ^a^	8.3 ± 0.3 ^a^
GPx_tot	84.4 ± 5.2 ^a^	79.1 ± 4.5 ^a^	74.2 ± 2.6 ^a^
GPx_Se	88.4 ± 5.3 ^a^	80.5 ± 2.8 ^a^	80.7 ± 2.8 ^a^
CAT	505.7 ± 54.2 ^a^	495.9 ± 39.7 ^a^	588.4 ± 76.2 ^a^
EROD	1.8 ± 0.3 ^ab^	2.1 ± 0.5 ^b^	0.9 ± 0.3 ^ac^
LPO	1.4 ± 0.2 ^a^	0.9 ± 0.1 ^b^	1.4 ± 0.1 ^a^

**Table 3:** Parasitological parameters of taxa recorded in hake (*Merluccius merluccius*) and parasite diversity indexes in the three different fishing ground of the Catalan coast. Values of prevalence *p* are presented as percentage and mean abundance (MA) are presented as mean ± standard error. Significant differences are indicated with asterisk comparing with north (GLMs, *p* < 0.05). A—adult, C—cavity, DT—digestive tract, G—gill, H—heart, I—intestine, L—liver, LIII—larva III, M—mesentery.

			North		Center		South		AVERAGE	
Length (cm)			30.83 ± 4.84		33.63 ± 3.89		31.29 ± 3.96		31.99 ± 4.40	
Condition Index (K)			0.70 ± 0.09		0.71 ± 0.03		0.71 ± 0.03		0.71 ± 0.06	
Parasite taxa	Stage	Infection site	P%	MA	P%	MA	P%	MA	P%	MA
Monogea	A	G	-	-	5	0.05 ± 0.22	7	0.07 ± 0.27	4	0.04 ± 0.19
Digenea	A	H	10	0.30 ± 0.98	10	0.15 ± 0.49	7	0.07 ± 0.27	9	0.18 ± 0.67
Cestoda	A	DT	35	0.50 ± 0.76	70 *	1.45 ± 1.50 *	50 *	1.07 ± 1.44 *	52	1.00 ± 1.30
Nematoda	LIII	C, M, L, DT	35	0.50 ± 0.83	35	0.65 ± 0.99	57	1.36 ± 2.06 *	41	0.80 ± 1.34
Acantocephala	A	I	-	-	-	-	14	0.28 ± 0.82	14	0.07 ± 0.43
Copepoda	A	G	-	-	-	-	7	0.07 ± 0.27	2	0.02 ± 0.14
Parasitic load			30		46		41			
Species richness (S)			5		4		6			
Shannon–Wiener diversity index (H)			1.42		0.94		1.28			
Evenness			0.82		0.64		0.6			

## Data Availability

Data related to the fishing locations and experimental tracks can be found in icatmar.cat. All other data can be available upon request.

## Supplementary Materials

The following supporting information can be downloaded at https://www.mdpi.com/article/10.3390/ani16010014/s1, Figure S1: Sorensen similarity indices for parasite assemblages; Figure S2: Macro-parasites identified in hake (*Merluccius merluccius*) in fishing grounds of the Catalan coast. (A) Adult of *Anthocotyle merluccii* Van Beneden & Hesse, 1863 (Monogenea), in toto and stained with Semichon’s acetocarmin, scale bar: 1 mm. (B) Adult of *Aporocotyle spinosicanalis* Williams, 1958 (Digenea), in toto and stained with Semichon’s acetocarmin, scale bar: 1 mm. (C) Male of *Acanthocephaloides propinquus* (Dujardin, 1845) Meyer, 1933 (Acanthocephala), in vivo, scale bar: 0.5 mm. (D) Females of *Lernaeocera* sp. (Copepoda) attached to gills, in vivo, scale bar: 5 mm. (E) Larva III of Anisakidae (Nematoda), in vivo, scale bar: 1 mm. (F) Adults of *Clestobothrium cras-siceps* (Rudolphi, 1819) Lühe, 1899 (Cestoda), in vivo, scale bar: 1 mm. (G) Plerocercoide larva of Tetraphyllidea (Cestoda), in toto and stained with Semichon’s acetocarmin, scale bar: 0.1 mm; Table S1: ANOVA test for the analyses of length, weight and gonadosomatic index of the studied hake individuals; Table S2: ANOVA test for the biochemical determinations analyzed for the studied hake individuals.

## Abbreviations

The following abbreviations are used in this manuscript:

PAHs	Polycyclic aromatic hydrocarbons
GES	Good environmental status
EU	European Union
PCBs	Polychlorinated biphenyls
DDT	Dichlorodiphenyltrichloroethane
TL	Total length
HSI	Hepatosomatic index
GSI	Gonadosomatic index
EDTA	Ethylenediaminetetraacetic acid
DTT	Dithiothreitol
EROD	Ethoxyresorufin-O-deethylase
ChE	Cholinesterase
AChE	Acetylcholinesterase
BChE	Butyrylcholinesterase
PrChE	Propionylcholinesterase
CE	Carboxylesterase
pNPA	p-nitrophenyl acetate
pNPB	p-nitrophenyl butyrate
αNA	α-naphthyl acetate
αNB	α-naphthyl butyrate
ASCh	Acetylthiocholine iodide
BSCh	Butyrylthiocholine iodide
PrSCh	Propionylthiocholine iodide
LPO	Lipid peroxidation
CAT	Catalase
GPX_tot	Glutathione peroxidase
GPx_Se	Selenium-dependent glutathione peroxidase
CHP	Cumene hydroperoxide
NADPH	Nicotinamide adenine dinucleotide phosphate
GR	Glutathione reductase
GSSG	Oxidized glutathione
GST	Glutathione S-transferase
MDA	Malondialdehyde
LDH	Lactate dehydrogenase
ANOVA	Analysis of variance
ANCOVA	Analysis of covariance
PCA	Principal component analyses
GLMs	Generalized Lineal Models
GSA	Geographical sub-areas
