# Supplementary material for: Characterizing the Health Status of European Hake (Merluccius merluccius) in Areas with Different Anthropic Impacts (NW Mediterranean Sea)
Source: Animals (Basel). 2025 Dec 19;16(1):14. doi: 10.3390/ani16010014 (PMC12785107; doi:10.3390/ani16010014)
Supplement: Supplementary file 1 [file animals-16-00014-s001.zip › animals-4008704-supplementary.pdf]

animals

MDPI

Figure S1. Sorensen similarity indices (qualitative and quantitative) for parasite assemblages between fishing ground on Catalan coast.

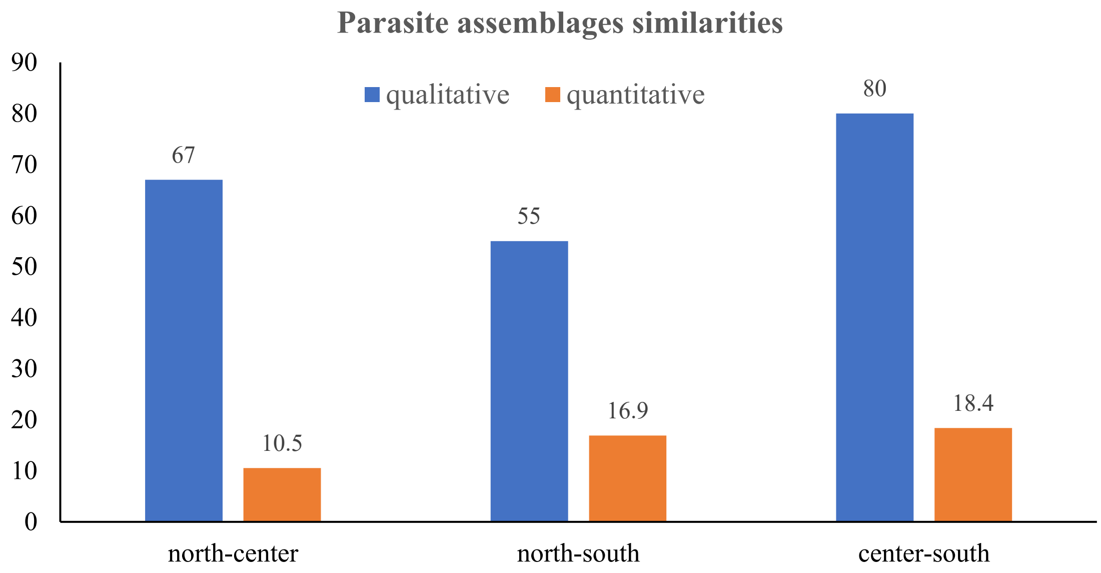

Figure S2. Macroparasites identified in hake (*Merluccius merluccius*) in fishing grounds off the Catalan coast. A) Adult of *Anthocotyle merluccii* Van Beneden & Hesse, 1863 (Monogenea), in toto and stained with Semichon´s acetocarmin, scale bar: 1 mm. B) Adult of *Aporocotyle spinosicanalis* Williams, 1958 (Digenea), in toto and stained with Semichon´s acetocarmin, scale bar: 1 mm. C) Male of *Acanthocephaloides propinquus* (Dujardin, 1845) Meyer, 1933 (Acanthocephala), in vivo, scale bar: 0.5 mm. D) Females of *Lernaeocera* sp. (Copepoda) attached to gills, in vivo, scale bar: 5 mm. E) Larva III of Anisakidae (Nematoda), in vivo, scale bar: 1 mm. F) Adults of *Clestobothrium crassiceps* (Rudolphi, 1819) Lühe, 1899 (Cestoda), in vivo, scale bar: 1 mm. G) Plerocercoide larva of Tetraphyllidea (Cestoda), in toto and stained with Semichon´s acetocarmin, scale bar: 0.1 mm.

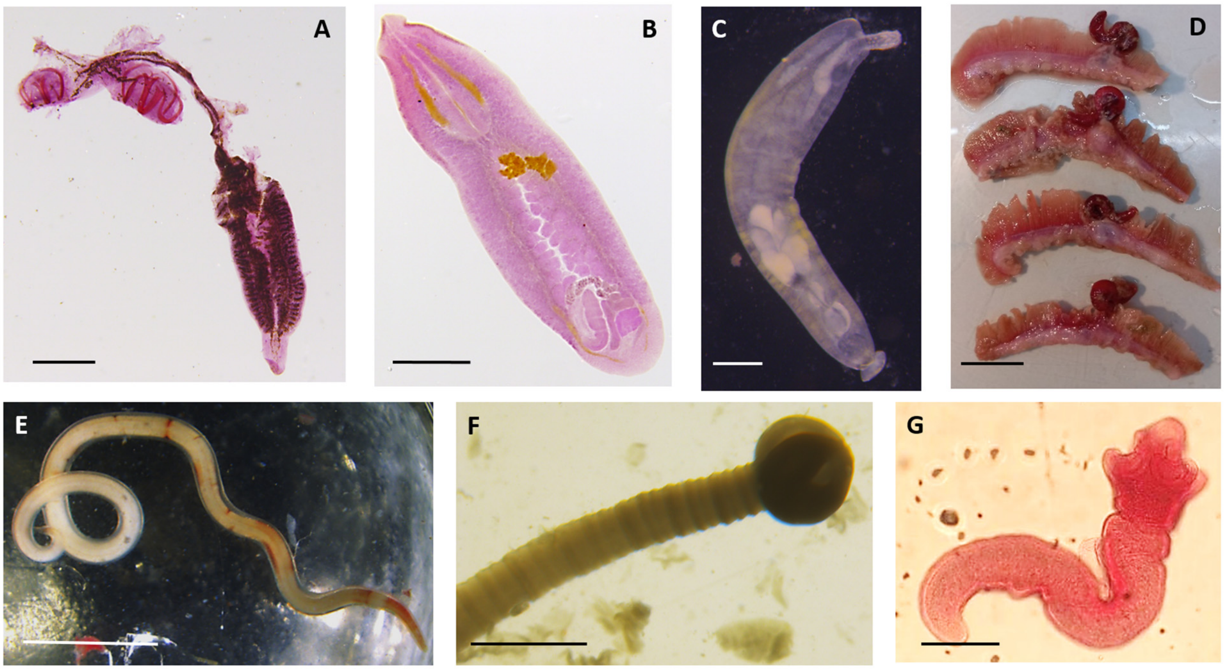

Table S1: ANOVA test for the analyses of length, weight and gonadosomatic index of the studied hake individuals.

Length

Source of Variation	df	Sum Sq	F	P-value
Between Groups	2	360.7	45.08	<0.0001
Within Groups	27	108.0		

Weight

Source of Variation	df	Sum Sq	F	P-value
Between Groups	2	82594	23.10	<0.0001
Within Groups	27	48280		

Gonadosomatic index

Source of Variation	df	Sum Sq	F	P-value
Between Groups	2	1.695	10.30	0.0005
Within Groups	27	2.220		

Table S2: ANOVA test for the biochemical determinations analyzed for the studied hake individuals.

Gonads

BChE

Source of Variation	df	Sum Sq	F	P-value
Between Groups	2	1.504	5.7956	0.00829
Within Groups	26	3.374		

PChE

Source of Variation	df	Sum Sq	F	P-value
Between Groups	2	1.5261	6.047	0.00721
Within Groups	25	3.1548		

Muscle

PChE

Source of Variation	df	Sum Sq	F	P-value
Intercept	1	446.94	15.3831	0.0005726
Between Groups	2	305.72	5.2612	0.0120606
Covariable (length)	1	168.92	5.8141	0.0232605
Within Groups	26	755.41		

pNPA

Source of Variation	df	Sum Sq	F	P-value
Between Groups	2	2.408	6.032	0.00683
Within Groups	27	5.388		

Brain

aNB

Source of Variation	df	Sum Sq	F	P-value
Between Groups	2	613.05	5.3606	0.01095
Within Groups	27	1543.88		

Liver

aNB

Source of Variation	df	Sum Sq	F	P-value
Intercept	1	24.0	0.1364	0.71517
Between Groups	2	1009.8	2.8695	0.07631
Covariable (length)	1	473.8	2.6931	0.11383
Within Groups	24	4222.8		0.14659

LPO

Source of Variation	df	Sum Sq	F	P-value

Between Groups	2	1.1166	6.4475	0.005321
Within Groups	26	2.2514		

EROD

Source of Variation	df	Sum Sq	F	P-value
Between Groups	2	1.0642	3.876	0.03478
Within Groups	24	3.2947		
